# Psychosocial Factors Mediate the Relationship Between Olfactory Function and Cognitive Frailty in Older Adults: A Structural Equation Modelling

**DOI:** 10.1111/ajag.70178

**Published:** 2026-05-09

**Authors:** Li‐Jun Chen, Chung‐Ying Lin, Chieh‐Ying Chou, Yu‐Chi Liao, Yi‐Ying Tsai, Esther En‐Shu Kuo, Fang‐Wen Hu

**Affiliations:** ^1^ Department of Internal Medicine National Cheng Kung University Hospital, College of Medicine, National Cheng Kung University Tainan Taiwan; ^2^ Institute of Allied Health Sciences, College of Medicine, National Cheng Kung University Tainan Taiwan; ^3^ Biostatistics Consulting Center, National Cheng Kung University Hospital, College of Medicine, National Cheng Kung University Tainan Taiwan; ^4^ Department of Public Health National Cheng Kung University, College of Medicine, National Cheng Kung University Tainan Taiwan; ^5^ Department of Occupational Therapy College of Medicine, National Cheng Kung University Tainan Taiwan; ^6^ Department of Family Medicine National Cheng Kung University Hospital, College of Medicine, National Cheng Kung University Tainan Taiwan; ^7^ Department of Psychology College of Medical and Health Science, Asia University Taichung Taiwan; ^8^ Center for Prevention and Treatment of Internet Addiction, Asia University Taichung Taiwan; ^9^ Clinical Psychology Center, Asia University Hospital Taichung Taiwan; ^10^ Department of Physical Therapy College of Medicine, National Cheng Kung University Tainan Taiwan; ^11^ School of Nursing, College of Nursing, Kaohsiung Medical University Kaohsiung Taiwan; ^12^ Center for Long‐Term Care Research, Kaohsiung Medical University Kaohsiung Taiwan; ^13^ Department of Medical Research Kaohsiung Medical University Hospital Kaohsiung Taiwan

**Keywords:** frailty, olfaction, resilience, social network, structural equation modeling

## Abstract

**Objective:**

With the trend of population ageing, cognitive frailty in older adults is an increasingly pressing public health issue. Identifying early‐stage risk factors has become a key priority for researchers with the goal of preventing disability. This study aimed to test a conceptual model and elucidate the pathways leading to cognitive frailty using Structural Equation Modelling (SEM).

**Methods:**

We conducted a cross‐sectional design with data from outpatient departments within a tertiary medical centre in southern Taiwan. Participants (*n* = 208) were aged 65 years or older and able to communicate independently with the researchers. Demographic factors, extant health conditions, and psychosocial factors were collected. Structural Equation Modelling (SEM) was used to construct a path model.

**Results:**

Olfactory function was significantly positively correlated with nutritional status and significantly negatively correlated with depressive symptoms, resilience, and cognitive frailty. Nutritional status was significantly negatively correlated with sarcopenia. Resilience was significantly positively correlated with social support but negatively correlated with sarcopenia. Sarcopenia was significantly positively correlated with cognitive frailty. Social support was significantly negatively correlated with cognitive frailty. Despite these associations, in this model, only one specific serial indirect pathway—via resilience and social support—demonstrated a statistically significant mediating effect between olfactory function and cognitive frailty.

**Conclusions:**

The findings suggest that the association between olfactory function and cognitive frailty may involve a limited, pathway‐specific serial mediation through resilience and social support. Clinically, this pathway may help to inform the early identification of older adults with olfactory impairment who may benefit from targeted supportive strategies aimed at strengthening resilience and social support.

## Practice Impact

This study demonstrates that olfactory dysfunction is a feasible early indicator of cognitive frailty. Incorporating olfactory function assessment and psychosocial evaluation into routine geriatric care enables timely, targeted interventions to enhance resilience and social support, thereby reducing cognitive frailty risk and improving healthy ageing outcomes.

## 1 Introduction

Dementia is a growing global health concern, with the World Health Organization estimating that over 55 million people were diagnosed with the condition as of 2023, and approximately 10 million new cases will occur annually [1]. The profound socioeconomic burden associated with dementia has shifted clinical and public health attention toward earlier stages of cognitive decline, highlighting the need to identify at‐risk populations where interventions may be more effective. Emerging evidence indicates that physical frailty and cognitive impairment often coexist and that their co‐occurrence is associated with a markedly increased risk of developing dementia compared with either condition alone [2]. In response to this, the International Academy on Nutrition and Aging and the International Association of Gerontology and Geriatrics proposed the concept of ‘cognitive frailty’ in 2013. Cognitive frailty, defined as the coexistence of mild cognitive impairment and physical frailty in the absence of dementia, represents a distinct geriatric syndrome along the continuum of cognitive ageing. Unlike established dementia, it is considered potentially reversible, providing a critical opportunity for early prevention [3].

Cognitive frailty is a multifactorial syndrome influenced by both non‐modifiable and modifiable determinants. While advanced age, lower educational attainment, and chronic comorbidities such as hypertension and diabetes are well‐established risk factors, growing evidence underscores the critical role of lifestyle and psychosocial factors [4]. Physical inactivity, depressive symptoms, lower resilience, and reduced social engagement have been consistently associated with the onset and progression of cognitive frailty [5, 6]. Importantly, unlike fixed demographic characteristics, many of these physical and psychosocial factors are potentially modifiable, highlighting cognitive frailty as a meaningful target for early intervention. Early identification and comprehensive management of these interrelated factors may help mitigate physical decline, preserve cognitive reserve, and potentially slow progression toward dementia.

In addition to commonly identified risk factors for cognitive frailty, olfactory dysfunction, including hyposmia and anosmia, is prevalent among older adults and has been recognised as an early indicator of neurodegenerative processes [7]. Previous studies have reported a significant association between olfactory impairment and dementia, with olfactory‐related neuropathological changes frequently observed prior to overt cognitive decline [8, 9]. Olfactory function also plays an important role in appetite regulation and dietary intake, and impairment in this sensory domain may contribute to nutritional inadequacy in older adults [10]. Reduced nutrient intake, together with age‐related inflammatory processes, has been linked to declines in muscle function [10], which are key components of sarcopenia and physical frailty. Taken together, these observations suggest that olfactory dysfunction may represent a potential early marker of cognitive frailty, linking sensory decline with broader functional vulnerability in later life.

Previous studies on cognitive frailty have primarily focused on the independent direct effects of various risk factors, with less exploration of their potential interactive effects and interconnected pathways. This study aimed to address this gap by considering olfactory function as an early‐stage factor in cognitive frailty. To achieve this, Structural Equation Modelling (SEM) was used to test a conceptual model and elucidate the pathways leading to cognitive frailty.

## 2 Methods

### 2.1 Study Design and Participants

A cross‐sectional study was conducted with potential participants identified from the outpatient departments of family medicine and geriatrics at a tertiary care medical centre in southern Taiwan. The inclusion criterion was individuals 65 years or older who were able to communicate independently with the researchers. The exclusion criteria were: (1) diagnosis of dementia; (2) cognitive impairment caused by neurological damage (i.e., stroke or traumatic brain injury) or psychotic disorder (i.e., schizophrenia); (3) olfactory impairment caused by sinonasal disease, viral infection or trauma; and (4) inability to walk independently using aids. The participants provided written informed consent, and the study was approved by the Institutional Review Board of the participating hospital.

### 2.2 Measurements and Procedures

Participants were recruited between April 2022 and January 2023. During the study period, a trained research nurse attended the outpatient departments from Monday to Friday to identify and recruit eligible participants. Prior to the commencement of outpatient clinics, the nurse screened consecutive outpatient appointment lists according to the predefined inclusion criteria. Potentially eligible individuals were approached during their scheduled outpatient visits and invited to participate in the study. Following the provision of written informed consent, participants' medical records were reviewed and face‐to‐face interviews were conducted in a private meeting room. Each assessment session lasted approximately 20–30 min and comprised sensory testing, physical performance tasks and questionnaire‐based assessments. Medical records provided information on demographic factors (age, sex, educational level and marital status) and chronic diseases. Interviews were conducted and included the following assessments: the Top International Biotech Smell Identification Test (TIBSIT), Short Form Mini Nutritional Assessment (MNA‐SF), sarcopenia, cognitive frailty, five‐item Geriatric Depression Scale (GDS‐5), Brief Resilience Scale (BRS), and Lubben Social Network Scale‐Revised (LSNS‐R).

#### 2.2.1 Top International Biotech Smell Identification Test (TIBSIT)

The Taiwan Smell Identification Test (TWSIT) was developed and successfully administered to the Taiwanese population. This was strongly correlated with the traditional Chinese version of the University of Pennsylvania Smell Identification Test (*r* = 0.87) [11]. However, for sanitation reasons, the commercialised TWSIT was redesigned as TIBSIT (Top International Biotech, Taipei, Taiwan). This is a ‘scratch‐and‐sniff’ form consisting of embedded fragrant microcapsules. The scents of the eight odorants were the same as in the TWSIT, and the format of the 16‐item questionnaire was identical. The participant completed the test without assistance from medical personnel by scratching the fragrant microcapsules with a pencil and smelling the fragrance released from the microcapsules. After the participants sniffed the odours, they answered the corresponding question to identify them. Each question contained two sub‐questions, one was a four‐item multi‐choice question where participants gain one point if the correct odorant is chosen from the four choices; the other was a three‐item question; ‘not detectable’, can smell nothing at all (0 points), ‘detectable, but not sure’, can smell something but unsure (1 point), and ‘detectable’, can smell and know exactly what it is (2 points). Thus, a normosmic participant gained one point for correctly choosing the odorant and an extra two points for choosing ‘detectable’ for a maximum score of three points per odorant. However, if the participant chose ‘detectable’ but the answer was incorrect, zero points were scored for this odorant. Hence, there were a total of 48 points for the 16 questions. For participants aged 55 years and older, a score over 40 points is normosmia, 12–29 points is hyposmia, under 11 points is anosmia [12].

#### 2.2.2 Short Form Mini Nutritional Assessment (MNA‐SF)

The original Mini Nutritional Assessment (MNA) comprises 18 items, which may limit its practicality for routine screening. To address this, Rubenstein et al. [13] developed a shortened version, the MNA‐SF, consisting of six items with a total score ranging from 0 to 14. Based on the score, individuals are categorised as well‐nourished (12–14), at risk of malnutrition (8–11), or malnourished (0–7). The MNA‐SF and MNA scores were strongly correlated (*r* = 0.95), with a sensitivity of 0.97, a specificity of 1.00, and a diagnostic accuracy of 0.98 for predicting undernutrition.

#### 2.2.3 Sarcopenia

Sarcopenia was defined as a low appendicular skeletal muscle mass (ASM) with either low muscle strength or low physical performance. Severe sarcopenia was defined as a low ASM with low muscle strength and low physical performance [13]. The Asian Working Group for Sarcopenia 2019 retained the previous definition of sarcopenia but revised the diagnostic criteria: low muscle strength is defined as handgrip strength < 28 kg for males and < 18 kg for females; criteria for low physical performance is Short Physical Performance Battery (SPPB) score ≤ 9; cutoffs for ASM index are Bioelectrical Impedance Analysis (BIA) < 7.0 kg/m^2^ in males and < 5.7 kg/m^2^ in females [14].

1. Handgrip strength was assessed using a handheld dynamometer (Jamar Hydraulic Dynamometer; J. A. Preston Corporation, New York, United of States). Using a handheld dynamometer to assess muscle strength has been shown to be reliable and valid in hospitalised older patients [15]. During the three‐test trials, participants remained seated with their arm resting on a table as they squeezed the dynamometer using an underhand grip with their dominant hand in the supinated position, exhaling while squeezing with maximal exertion. The highest value of the three‐test trials performed was included.
2. Short Physical Performance Battery (SPPB) assesses physical performance using three tests: balance, strength and gait. Balance was assessed based on the ability to stand upright in three different positions for 10 s each: feet together, with one foot partially forward and with one foot forward. Strength and gait were first evaluated by the ability to perform the tasks of getting up and sitting on a chair five consecutive times, performing the walking speed test (4 metres), and the time the participant took to complete the tasks. Each test was scored from 0 points (inability to perform the task) to 4 points (best test performance), with the total score ranging from 0 points (worst performance) to 12 points (best performance) [16]. The SPPB has been shown to have predictive validity, with a gradient of risk for mortality, nursing home admission and disability [16].
3. Bioelectrical Impedance Analysis (BIA): The ASM index (ASM/height^2^) is determined using BIA (InBody 270; InBody Corporation, Seoul, Korea). This BIA model uses eight electrodes positioned at each hand and foot and enables multifrequency impedance measurements of the arms, trunk and legs. According to the InBody 270 manufacturer's guidelines, the arms of the subjects were kept straight, did not touch the body, their legs were kept apart, and their heels were placed on the rear sole electrodes. Four fingers wrapped the surface of the bottom‐hand electrode, and the thumb was placed on the oval electrode. Subjects were not touched by the examiner during the test.

#### 2.2.4 Cognitive frailty

Cognitive frailty was defined as a combination of physical frailty and Mild Cognitive Impairment (MCI).

1. Fried's frailty phenotype assesses physical frailty through five indicators: unintentional weight loss (lost > 10 pounds unintentionally in the last year), weakness (handgrip strength ≤ 29–32 kg for males [stratified by body mass index classifications] and ≤ 17–21 kg for females [stratified by body mass index classifications]), self‐reported exhaustion, slowness (4 m walk ≥ 7 s [male height ≤ 173 cm, female height ≤ 159 cm] or ≥ 6 s [male height > 173 cm, female height > 159 cm]), and low physical activity (energy expenditure < 383 kcal per week for males and < 270 kcal per week for females). Physical frailty was identified as the presence of three or more of the indicators [17].
2. Saint Louis University Mental Status (SLUMS) assesses MCI using 11 questions, 10 of which are scored. This assessment tests for orientation, memory, attention, and executive functions. Depending on the question, answers score from 0 to 5 points. Final SLUMS scores range from 0 to 30. There are two scoring structures depending on the level of education of the participant. For participants who had completed high school, the SLUMS score structure was: 27–30 (normal), 21–26 (MCI), and < 20 (dementia). For subjects who had not completed high school, the SLUMS score structure was: 25–30 (normal), 20–24 (MCI), and < 19 (dementia) [18].

#### 2.2.5 Five‐item Geriatric Depression Scale (GDS‐5)

The severity of depressive symptoms was assessed using the GDS‐5. This scale consists of five items, with responses to the first item rated as 0 = yes and 1 = no, and responses to the following four items rated as 0 = no and 1 = yes. A high GDS‐5 score indicates high severity of depressive symptoms [19]. This scale had satisfactory internal consistency (*α* = 0.83–0.90), a sensitivity of 0.94, specificity of 0.81, positive predictive value of 0.81, negative predictive value of 0.94, positive likelihood ratio of 4.92 and negative likelihood ratio of 0.07 [20].

#### 2.2.6 Brief Resilience Scale (BRS)

The BRS was developed to measure the ability to bounce back or recover from stress. The participants were asked to indicate how well each statement described their behaviour and actions on a five‐point Likert scale, ranging from ‘1’, does not describe me at all to ‘5’, describes me very well. As Item 2 (I have a hard time making it through stressful events), Item 4 (It is hard for me to snap back when something bad happens) and 6 (I tend to take a long time to get over setbacks in my life) were reverse coded, the collected data were recoded prior to analysis. Higher scores indicated higher resilience. The Chinese version of the BRS has satisfactory internal consistency (α = 0.70) and good construct validity [21].

#### 2.2.7 Lubben Social Network Scale‐Revised (LSNS‐R)

The LSNS‐R is a revised version of the original tool (LSNS) from 1988. This scale is designed to measure social isolation in older adults by asking about the perceived social support received from family, friends, and mutual supports. It consists of 12 items that measure the size, closeness, and frequency of contact of the social network. The scores for each question range from 0 to 5, with 0 indicating minimal social integration and 5 indicating substantial social integration. The total score ranged from 0 to 60, with higher scores indicating a greater level of social support and a lower risk of isolation [22].

### 2.3 Data Analysis

The data were first analysed using descriptive statistics to summarise demographic factors, extant health conditions and psychosocial factors. Zero‐order Pearson correlations were conducted to determine the bivariate correlations between extant health conditions and psychosocial factors. Finally, SEM with a diagonally weighted least squares estimator was used to examine the proposed conceptual model and an alternative model (i.e., a reduced model, please see details below). In both models, path models were examined to satisfy the principle of parsimony in statistical analyses. Specifically, the comparative fit index (CFI), Tucker‐Lewis index (TLI), root mean square error of approximation (RMSEA) and standardised root mean square residual (SRMR) were used to examine the fit of the data model. A CFI and TLI > 0.9, together with RMSEA and SRMR < 0.08, indicate a satisfactory fit. However, an RMSEA < 0.10 can also be considered as acceptable. Based on the fitted model, the paths in the proposed model were examined to determine whether they were significant. In the proposed model, age, sex and education were adjusted as covariates, and mediation effects were further examined using bootstrapping methods with 1000 resamples. Apart from testing the conceptual model, we planned to test an alternative model based on the conceptual model. That is, based on the findings of the conceptual model, those nonsignificant paths would be removed to become a reduced model to compare with the conceptual model. The SEM was analysed using the *lavaan* package in R software [23], and the other analyses were performed using SPSS 20.0 (IBM. Corp.: Armonk, NY).

## 3 Results

The study cohort included 208 older adults (mean (standard deviation [SD]) age = 75.66 [6.70] years; range, 65–94 years), with more females (*n* = 120; 58%). More than 40% of the participants (*n* = 91; 44%) had an educational level of primary school or below and almost three‐quarters (*n* = 151; 73%) were married. Among the 208 participants, 74 (36%) were classified as cognitive frailty. The mean SLUMS score for the total sample was 25.1 (SD = 4.1). Based on standard SLUMS thresholds, 120 participants (58%) were classified as having normal cognitive function, and 76 participants (37%) scored within the range for mild cognitive impairment. Twelve participants (6%) scored within the dementia range; however, they were retained in the analysis because none had a formal clinical diagnosis of dementia and all demonstrated sufficient functional capacity to complete study procedures. Table 1 shows the extant health conditions (i.e., chronic disease, olfactory function, MNA‐SF, and sarcopenia) and psychosocial factors (i.e., GDS‐5, BRS, and LSNS‐R) of the participants.

**TABLE 1:** Participants' characteristics (*n* = 208).

Characteristics	Mean (SD)/*n* (%)
Demographics factors	
Age; Mean (SD)	75.66 (6.70)
Sex	
Female	120 (58)
Male	88 (42)
Educational level	
Illiterate	24 (12)
Primary school	67 (32)
Junior high school	13 (6)
Senior high school	56 (27)
Undergraduate	42 (20)
Postgraduate	6 (3)
Marital status	
Married	151 (73)
Divorced or widowed	57 (27)
Extant health conditions	
Chronic disease	
Hypertension	128 (62)
Hyperlipidaemia	103 (50)
Diabetes mellitus	95 (46)
Chronic kidney disease	45 (22)
Cardiovascular disease	39 (19)
Hepatic disease	27 (13)
Lung disease	17 (8)
Thyroid disease	17 (8)
Olfactory function	
Normosmia	121 (58)
Hyposmia	83 (40)
Anosmia	4 (2)
MNA‐SF	
Normal	172 (83)
Malnutrition	36 (17)
Sarcopenia	
Normal	139 (67)
Sarcopenia	20 (10)
Severe sarcopenia	47 (23)
Psychosocial factors	
Depressive symptom	39 (19)
BRS; Mean (SD)	18.20 (3.91)
LSNS‐R; Mean (SD)	25.13 (10.05)

Abbreviations: BRS, Brief Resilience Scale; LSNS‐R, Lubben Social Network Scale‐Revised; MNA‐SF, Short Form Mini Nutritional Assessment.

Table 2 shows the zero‐order correlations between the study variables. All correlations were statistically significant, except for the marginally significant correlation between MNA‐SF and olfactory function (*r* = 0.125; *p* = 0.07). Specifically, cognitive frailty was positively correlated with sarcopenia (*r* = 0.382) and GDS‐5 (*r* = 0.209), and negatively correlated with MNA‐SF (*r* = −0.217), BRS (*r* = −0.355), LSNS‐R (*r* = −0.485), and olfactory function (*r* = −0.558).

**TABLE 2:** Pearson correlations between the present health conditions and psychosocial factors in the conceptual model.

	*r* (*p*)						
	Olfactory function	MNA‐SF	Sarcopenia	Cognitive frailty	GDS‐5	BRS	LSNS‐R
Olfactory function	—						
MNA‐SF	0.125 (0.07)	—					
Sarcopenia	−0.262 (< 0.001)	−0.336 (< 0.001)	—				
Cognitive frailty	−0.558 (< 0.001)	−0.217 (< 0.001)	0.382 (< 0.001)	—			
GDS‐5	−0.206 (0.003)	−0.269 (< 0.001)	0.154 (0.03)	0.209 (0.002)	—		
BRS	0.389 (< 0.001)	0.281 (< 0.001)	−0.327 (< 0.001)	−0.355 (< 0.001)	−0.445 (< 0.001)	—	
LSNS‐R	0.375 (< 0.001)	0.224 (< 0.001)	−0.429 (< 0.001)	−0.485 (< 0.001)	−0.430 (< 0.001)	0.550 (< 0.001)	—

Abbreviations: BRS, Brief Resilience Scale; GDS‐5, Five‐item Geriatric Depression Scale; LSNS‐R, Lubben Social Network Scale‐Revised; MNA‐SF, Short Form Mini Nutritional Assessment.

**FIGURE 1:**
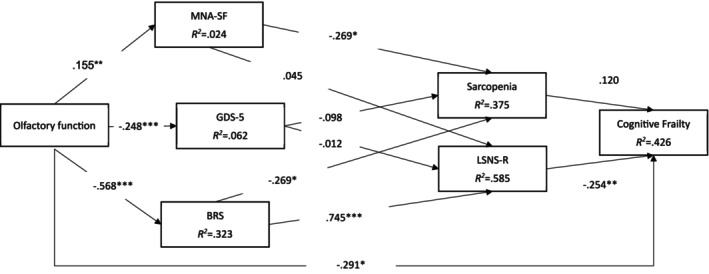
Results of the conceptual model. *p < 0.05; **p < 0.01; ***p < 0.001; age, sex, and education were controlled; Fit statistics: Comparative fit index = 0.957; Tucker‐Lewis index = 0.906; root mean square error of approximation = 0.092 (90% CI = 0.062, 0.124); standardised root mean square residual = 0.068. *χ* ^2^ (df) = 49.452 (18); *p* < 0.001. BRS, Brief Resilience Scale; GDS‐5, Five‐item Geriatric Depression Scale; LSNS‐R, Lubben Social Network Scale‐Revised; MNA‐SF, Short Form Mini Nutritional Assessment.

The proposed conceptual model adjusted for age, sex and education showed a satisfactory fit to the data (CFI = 0.957, TLI = 0.906, RMSEA = 0.092, and SRMR = 0.068) (Figure 1).

Moreover, most of the proposed paths had significant direct correlations, except for GDS‐5 to sarcopenia (standardised coefficient = −0.098; *p* = 0.45), LSNS‐R (standardised coefficient = −0.012; *p* = 0.93), MNA‐SF to LSNS‐R (standardised coefficient = −0.045; *p* = 0.68) and sarcopenia to cognitive frailty (standardised coefficient = 0.120; *p* = 0.28). Regarding the variance explained by the studied variables, *R* ^2^ was 0.426 for cognitive frailty, 0.375 for sarcopenia, 0.585 for the LSNS‐R, 0.024 for the MNA‐SF, 0.062 for the GDS‐5 and 0.323 for the BRS. In addition, none of the mediation effects were significant, except for the path from olfactory function to cognitive frailty via BRS and LSNS‐R (standardised coefficient = −0.108; *p* = 0.02) (Table 3).

**TABLE 3:** Mediation effects in the conceptual model.

	Unstand. coeff.	Bootstrap SE	*p*	Stand. coeff.
Figure 1 (conceptual model without removing any paths)				
Olfactory function➔ MNA‐SF➔ sarcopenia➔ cognitive frailty	−0.005	0.006	0.41	−0.005
Olfactory function➔ GDS‐5➔ sarcopenia ➔ cognitive frailty	0.003	0.006	0.65	0.003
Olfactory function➔ BRS➔ sarcopenia ➔ cognitive frailty	−0.018	0.015	0.25	−0.018
Olfactory function➔MNA‐SF➔ LSNS‐R➔ cognitive frailty	−0.002	0.010	0.87	−0.002
Olfactory function➔ GDS‐5➔ LSNS‐R➔ cognitive frailty	−0.001	0.025	0.98	−0.001
Olfactory function➔ BRS➔ LSNS‐R➔ cognitive frailty	−0.105	0.044	0.02	−0.108
Figure 2 (conceptual model with nonsignificant paths from Figure 1 removed)				
Olfactory function➔ BRS➔ LSNS‐R➔ cognitive frailty	−0.123	0.032	< 0.001	−0.126

*Note:* Both models adjusted age, sex, and education.

Abbreviations: BRS, Brief Resilience Scale; GDS‐5, Five‐item Geriatric Depression Scale; LSNS‐R, Lubben Social Network Scale‐Revised; MNA‐SF, Short Form Mini Nutritional Assessment; SE, standard error; Stand. coeff., standardised coefficient; Unstand. coeff, unstandardised coefficient.

**FIGURE 2:**
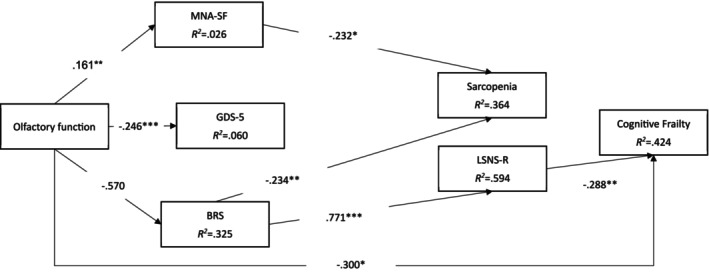
Results of the conceptual model with non‐significant paths removed. *p < 0.05; **p < 0.01; ***p < 0.001; age, sex, and education were controlled; Fit statistics: Comparative fit index = 0.957; Tucker‐Lewis index = 0.913; root mean square error of approximation = 0.089 (90% CI = 0.059, 0.120); standardised root mean square residual = 0.068. *χ* ^2^ (df) = 49.839 (19); *p* < 0.001. BRS, Brief Resilience Scale; GDS‐5, Five‐item Geriatric Depression Scale; LSNS‐R, Lubben Social Network Scale‐Revised; MNA‐SF, Short Form Mini Nutritional Assessment.

The alternative reduced model, which also adjusted for age, sex and education, showed similar fit indices to the proposed conceptual model (CFI = 0.957, TLI = 0.913, RMSEA = 0.089 and SRMR = 0.068), though slightly better (Figure 2). Moreover, the significant paths shown in the proposed conceptual model (Figure 1) remained significant in the reduced model (Figure 2); the solely significant mediation also remained significant in the reduced model (standardised coefficient = −0.123; *p* < 0.001) (Table 3).

## 4 Discussion

This study aimed to test a conceptual model elucidating the mechanisms linking olfactory impairment to cognitive frailty. Our findings revealed several significant associations among olfactory function, nutritional status, resilience, social support, sarcopenia and cognitive frailty. Nonetheless, only one specific serial indirect pathway—via resilience and social support—demonstrated a statistically significant mediating effect between olfactory function and cognitive frailty. Although nutrition and depression were examined as potential mediators, their explanatory power was minimal, indicating a limited contribution to the overall model. Overall, these results highlight that the mediating role of psychosocial factors is restricted and pathway‐specific, rather than broad or generalised.

### 4.1 Olfactory Function

This study found that olfactory function was positively correlated with nutritional status but negatively correlated with depressive symptoms. Accumulating evidence indicates that a reduction in appetite and nutrient intake, particularly a marked decline in protein consumption, leads to malnutrition due to hyposmia [10]. Furthermore, olfactory transmission involves a neural pathway that includes the olfactory bulb, primary olfactory cortex, limbic system, and orbitofrontal cortex. The limbic system is closely associated with emotion processing. Previous studies have reported a significant correlation between olfactory dysfunction and depression [24].

This study revealed a negative correlation between olfactory function and resilience, a finding that is contrary to common clinical expectations, as better olfactory function is generally assumed to support coping and adaptive capacities. Interestingly, previous research on protective factors, such as Sensory Processing Sensitivity and Attention Awareness, has demonstrated that individual traits can buffer vulnerabilities and enhance resilience [25]. By analogy, our counter‐intuitive finding may reflect the influence of residual confounding factors or complex neurobehavioral mechanisms that have yet to be fully elucidated.

With respect to olfaction, once olfactory information reaches the prefrontal cortex, it undergoes further processing and encoding, leading to the formation of olfactory memories that can elicit diverse emotional responses, both positive and negative. The prefrontal cortex is critically involved in resilience‐related functions, including emotional regulation, stress modulation and attenuation of fear responses originating from the amygdala [26]. Nonetheless, while olfactory function and resilience are associated with the prefrontal cortex, the specific mechanisms underlying their relationship remain unclear. Future studies could employ neuroimaging to explore prefrontal activity during olfactory and resilience‐related tasks, or utilise comprehensive behavioural assessments to better characterise this association.

### 4.2 Nutritional Status

Malnutrition has been linked to a decline in cellular metabolism, including weight loss, decreased muscle strength and reduced muscle mass, potentially contributing to the development of sarcopenia in older adults. Chang et al. [27] have reported a higher risk of sarcopenia among individuals with malnutrition. However, in our model, nutritional status showed limited explanatory power and did not exert a significant mediating effect between olfactory function and cognitive frailty.

### 4.3 Resilience

The results revealed a significant positive correlation between resilience and social support and a negative correlation between resilience and sarcopenia. Social support, characterised by the provision of care and concern, has consistently been linked to enhanced resilience in individuals facing adversity. Wang et al. [28] demonstrated a significant positive correlation between social support and resilience among older adults. Notably, resilience plays a crucial role in buffering the detrimental effects of stressful events, thereby promoting physical recovery and delaying the onset of frailty and sarcopenia. Furthermore, Cesari et al. [29] found that higher levels of resilience can mitigate cellular senescence and enhance skeletal muscle metabolism, ultimately reducing the incidence of sarcopenia.

### 4.4 Sarcopenia

Characterised by a decline in skeletal muscle function and activity, sarcopenia was significantly correlated with cognitive frailty in older adults. This condition, coupled with decreased physical activity, increases the risk of cognitive decline, as evidenced in studies conducted by Inoue et al. [30] Moreover, the role of sarcopenia as a significant risk factor for cognitive frailty underscores its contribution to the multifaceted interplay between physical and cognitive health in older adults.

### 4.5 Social Support

Social support was significantly and negatively correlated with cognitive frailty in older adults. Those with poor social support, social isolation and loneliness were at increased risk of reduced physical activity, malnutrition and depression, all of which can contribute to cognitive decline and physical frailty [4]. Xie et al. [31] further supported this finding, reporting a significant negative correlation between social participation, social support, and cognitive frailty.

The SEM results suggested a direct effect of olfactory function on cognitive frailty, and a statistically significant serial indirect effect via resilience and social support. Although further studies are warranted to elucidate the specific correlation between olfactory function and resilience, it is evident that higher levels of resilience facilitate better adaptation to environmental changes, increase participation in social activities, and improve emotional regulation and interpersonal relationships in older adults. On the other hand, good social support fosters opportunities for social engagement, which in turn has a protective effect on cognitive function and can boost physical activity levels, thereby mitigating the progression of cognitive frailty [31]. Taken together, these findings highlight the complex interplay between olfactory function, psychosocial factors—particularly resilience and social support—and cognitive frailty in older adults, emphasising the importance of addressing psychosocial domains to support healthy ageing.

However, this study has some limitations. First, the cross‐sectional design precludes any inference of causality between the examined factors. Consequently, the findings from the SEM should be interpreted as indicative of associations and hypothesis‐generating rather than causal. Future research employing longitudinal designs is warranted to clarify the causal relationships among these variables. Second, the generalisability of the findings is limited by the recruitment of older adults from the outpatient departments of a tertiary care medical centre. This recruitment strategy may have introduced selection bias by under‐representing more frail or institutionalised older adults. Consequently, the study sample predominantly reflects relatively healthier, community‐dwelling individuals, and the findings should therefore be interpreted with caution when extrapolated to frailer populations or those residing in long‐term care settings. In addition, participants were recruited from outpatient departments using a consecutive sampling approach, which may also limit the generalisability of the findings and introduce potential selection bias, as older adults who did not attend outpatient clinics during the study period were not represented. Third, participants who scored within the dementia range on the SLUMS were retained in the analysis despite the absence of a formal clinical diagnosis of dementia. Although all such participants demonstrated sufficient functional capacity to complete study procedures, their inclusion may have influenced the estimated prevalence of cognitive frailty. Fourth, we only examined path models using observed variables as we intended to satisfy the principle of parsimony. However, this practice does not consider measurement modelling in the SEM and may be biased by measurement errors. Therefore, the present study's findings need to be interpreted with caution. Fifth, there is a conceptual and measurement overlap between sarcopenia and cognitive frailty, as sarcopenia shares components with the physical frailty criteria used to operationalise cognitive frailty, which may have contributed to an overestimation of their association. However, both constructs were defined according to widely accepted consensus criteria, and removing physical components would compromise the established definitions and limit comparability with prior studies. Future research may explore alternative operationalisations or longitudinal designs to further disentangle the shared and distinct mechanisms underlying these conditions. Finally, this study was conducted during the COVID‐19 pandemic, a period during which olfactory function may have been affected by viral exposure. In Taiwan, outpatient services implemented a symptom diversion policy: individuals with COVID‐19 related symptoms, fever or a positive COVID‐19 test were required to seek care in the emergency department and were not permitted to attend routine outpatient clinics. However, not all patients attending outpatient clinics were systematically tested for COVID‐19, and it is possible that individuals with very mild or asymptomatic infections were included. Therefore, although participants with overt COVID‐19 symptoms were largely excluded, the potential inclusion of individuals with mild or asymptomatic infection may have influenced olfactory measurements and should be considered when interpreting the findings.

## 5 Conclusions

While previous studies have predominantly focused on the impact of physiological and physical functions on cognitive frailty, this study highlighted the role of olfactory function as an early‐stage factor. Our findings indicated that a specific psychosocial pathway, via resilience and social support, may mediate the association between olfactory function and cognitive frailty. Rather than reflecting broad psychosocial determinants, these results underscored the importance of pathway‐specific mechanisms. Building on these insights, future research could further explore the interplay between olfactory function and psychosocial factors. Such knowledge may help inform targeted supportive strategies for older adults with olfactory impairment, potentially contributing to the prevention of cognitive frailty and promoting healthy ageing.

## Funding

This study was supported by a grant from the National Science and Technology Council (NSTC 112‐2314‐B‐037‐132‐MY3).

## Disclosure

No AI tools were used in data analysis or scientific interpretation.

## Ethics Statement

The participants provided written informed consent and the study was approved by the Institutional Review Board of the participating hospital (IRB No. A‐ER‐110‐329).

## Conflicts of Interest

The authors declare no conflicts of interest.

## Data Availability

The data that support the findings of this study are available from the corresponding author upon reasonable request.
